# Supplementary material for: Aglycosylated antibody-producing mice for aglycosylated antibody-lectin coupled immunoassay for the quantification of tumor markers (ALIQUAT)
Source: Commun Biol. 2020 Oct 30;3:636. doi: 10.1038/s42003-020-01363-9 (PMC7599229; doi:10.1038/s42003-020-01363-9)
Supplement: Supplementary file 1 — Supplementary Information [file 42003_2020_1363_MOESM1_ESM.pdf]

# Supplementary Information

**Aglycosylated antibody-producing mice for aglycosylated antibody-lectin coupled immunoassay for the quantification of tumor markers (ALIQUAT)**

Nan-Ee Lee[1,3,6], Sun Hee Kim[1,6], Dae-Yeul Yu[1], Eui-Jeon Woo[2,3], Myung-il Kim[4], Gi-Sang Seong[4], Sun Min Lee[5], Jeong-Heon Ko[1,3,*], Yong-Sam Kim[1,3,*]

[1]Genome Editing Research Center, [2]Disease Target Structure Research Center, KRIBB, Daejeon 34141, Republic of Korea; [3]Department of Bio-molecular Science, Korea University of Science and Technology (UST), Daejeon 34113, Republic of Korea; [4]Bioneer, Daejeon 34302, Republic of Korea; [5]Departments of Laboratory Medicine, Pusan National University Yangsan Hospital and School of Medicine, Gyeongnam, Republic of Korea

[6]These authors contributed equally

* Corresponding authors

**Supplementary Figures 1-10**

**Supplementary Tables 1-3**

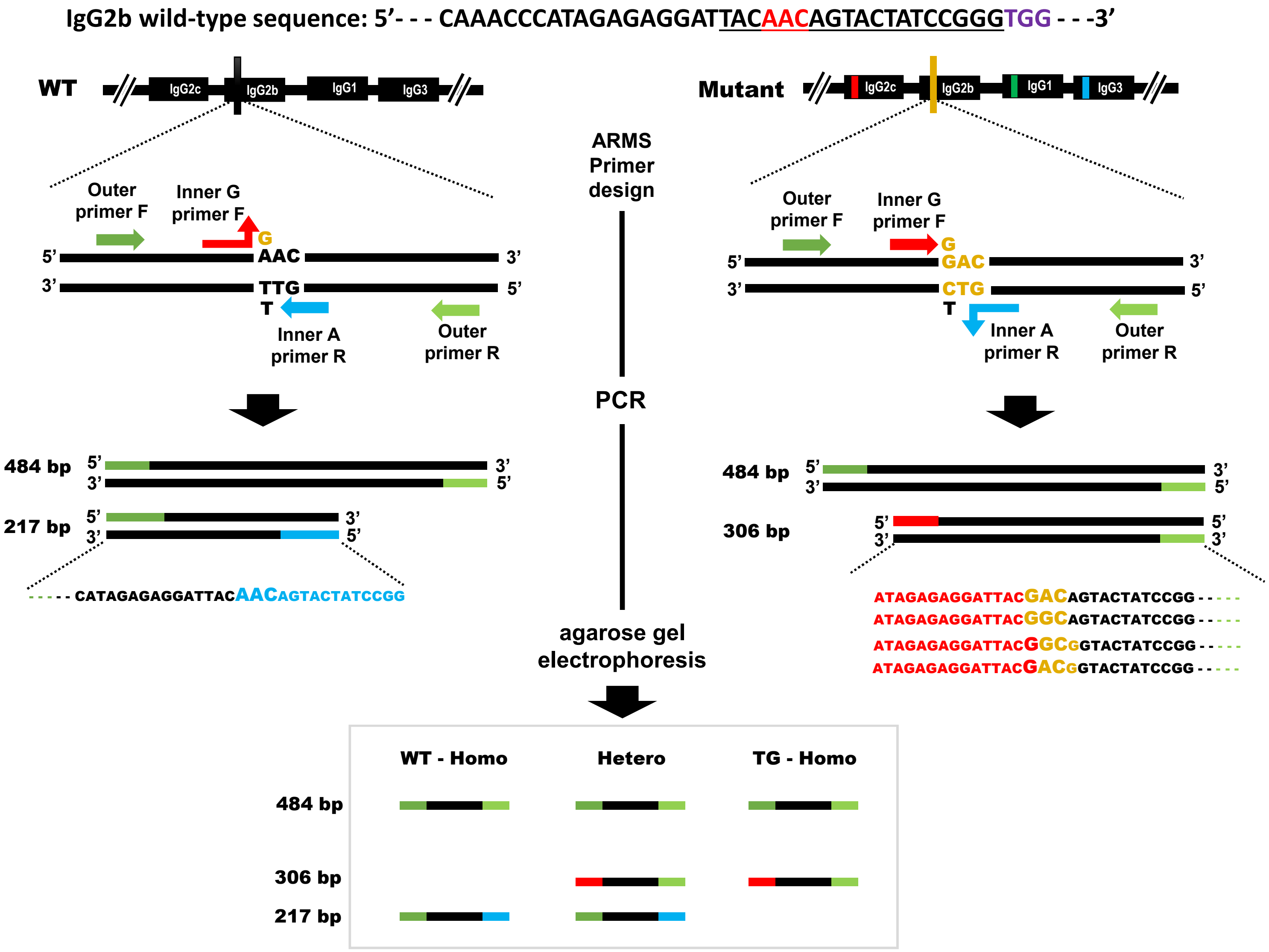


**Supplementary Figure 1. Scheme for the screening of base-edited mutant mice using an amplification refractory mutation system (ARMS).** Primers were designed to newly generate a 306-bp band on gels in a mutant gene, while a 217-bp band shown in a wild-type (WT) mice disappears. Accordingly, a mono-allelic mutation simultaneously renders both the 306- and 217-bp bands. Target and primer sequences in this scheme are exemplified for IgG2b, but other than those, the underlying scheme is identical for the screening of IgG2c and IgG3 mutant mice.

Ighg2c wild-type sequence: 5'---GAGAGGATTACAACAGTACTCTCCGGGTGGTCAGTGCC---3'

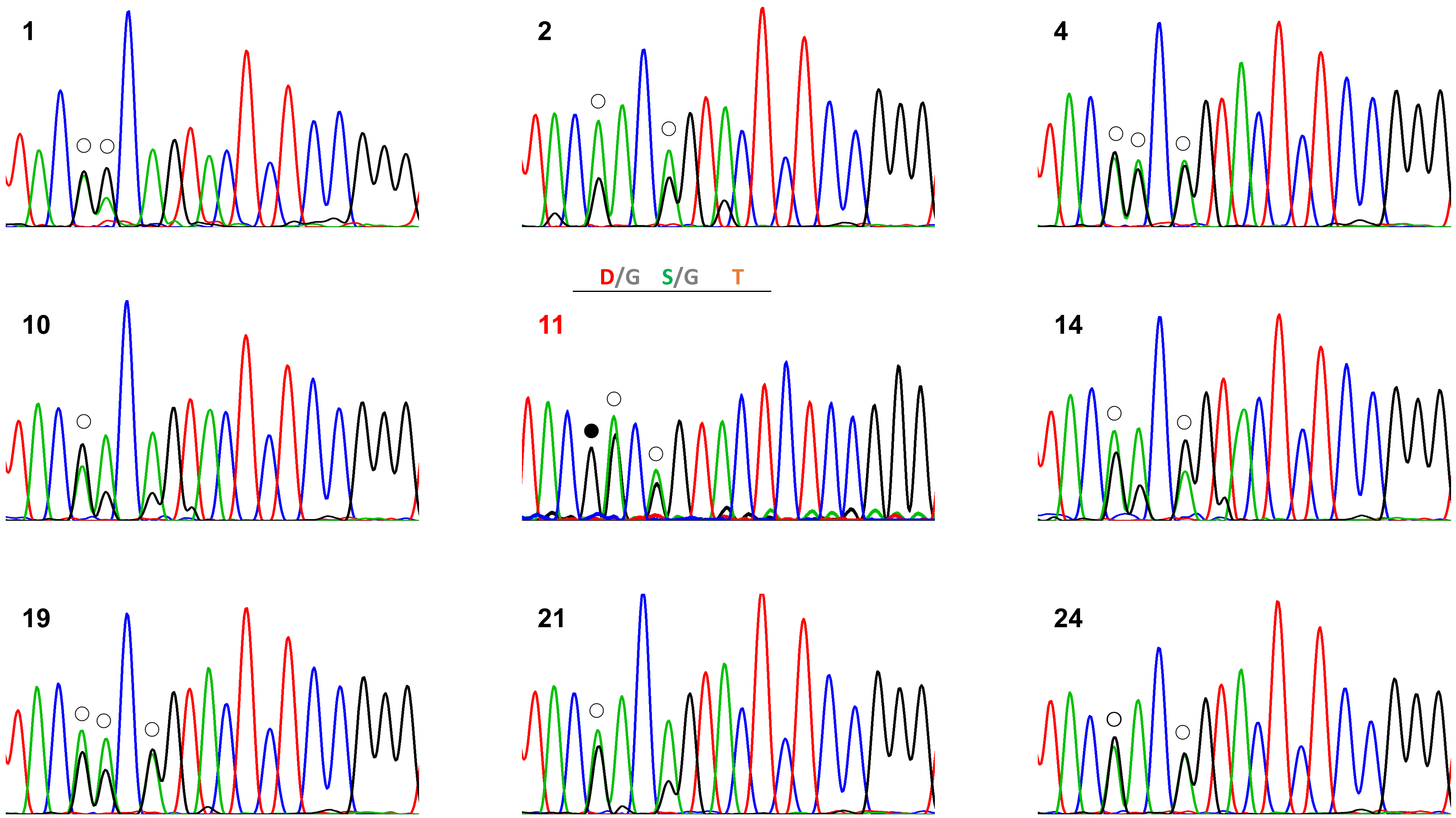


**Supplementary Figure 2a. Sanger sequencing analysis of Ighg2c genes of nine pups screened by ARMS after adenine base editing procedures.** Mono- and bi-allelic mutations were marked with a closed and an open circle, respectively. The modified amino acid sequences were noted for pup#11.

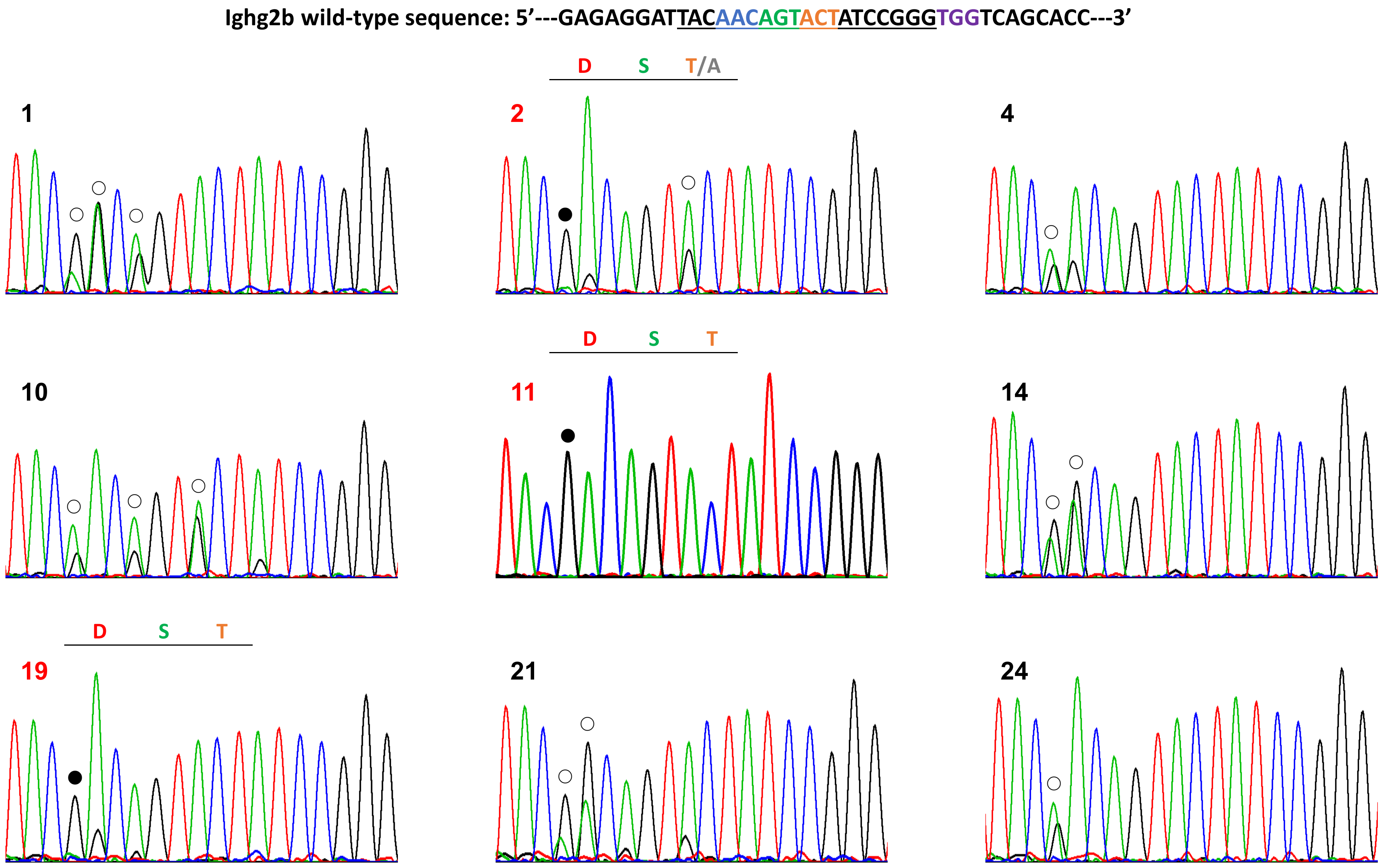


**Supplementary Figure 2b. Sanger sequencing analysis of Ighg2b genes of nine pups screened by ARMS after adenine base editing procedures**. Mono- and bi-allelic mutations were marked with a closed and an open circle, respectively. The modified amino acid sequences were noted for pup#2, 11, and 19.

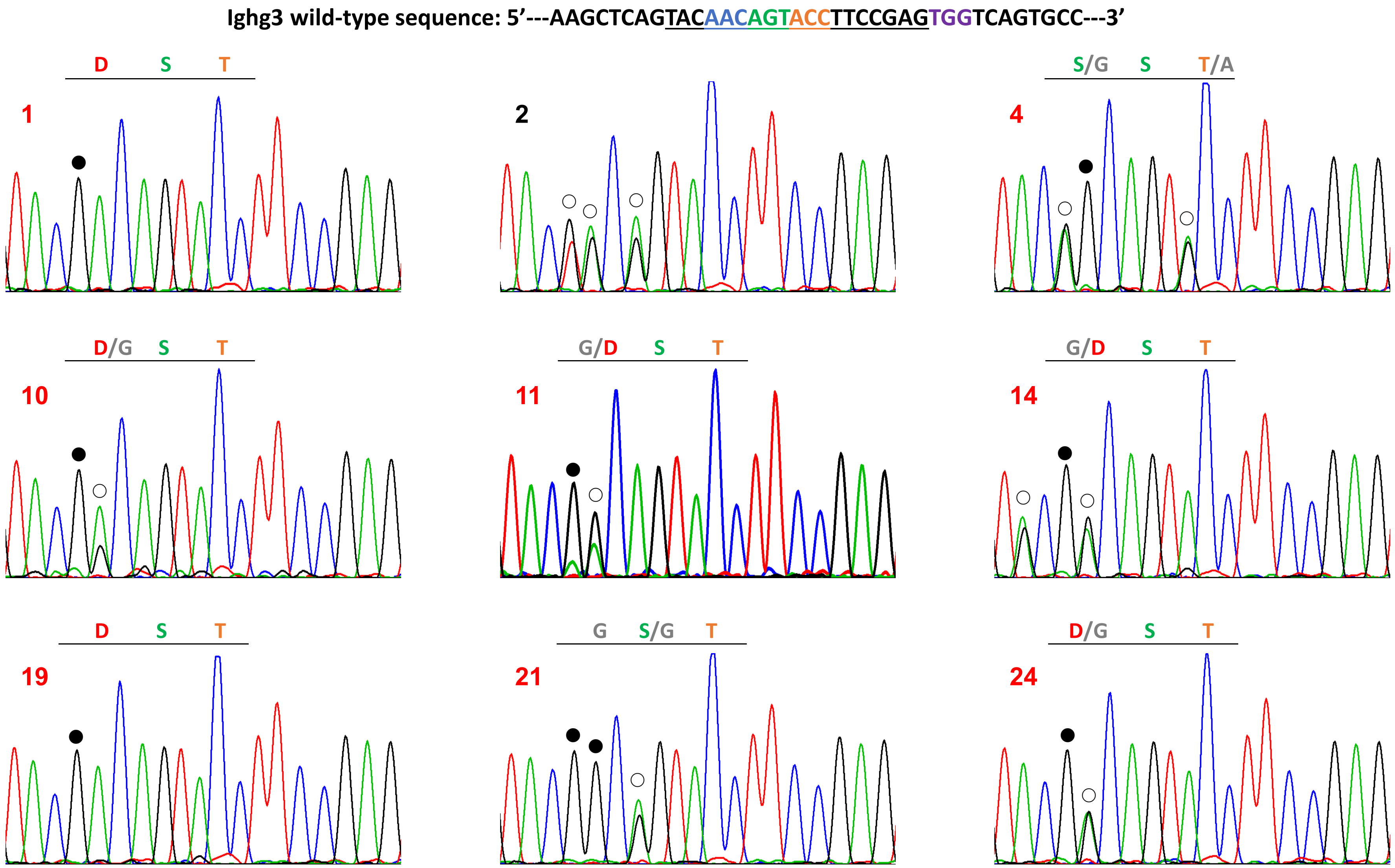


**Supplementary Figure 2c. Sanger sequencing analysis of Ighg2c genes of nine pups screened by ARMS after adenine base editing procedures.** Mono- and bi-allelic mutations were marked with a closed and an open circle, respectively.

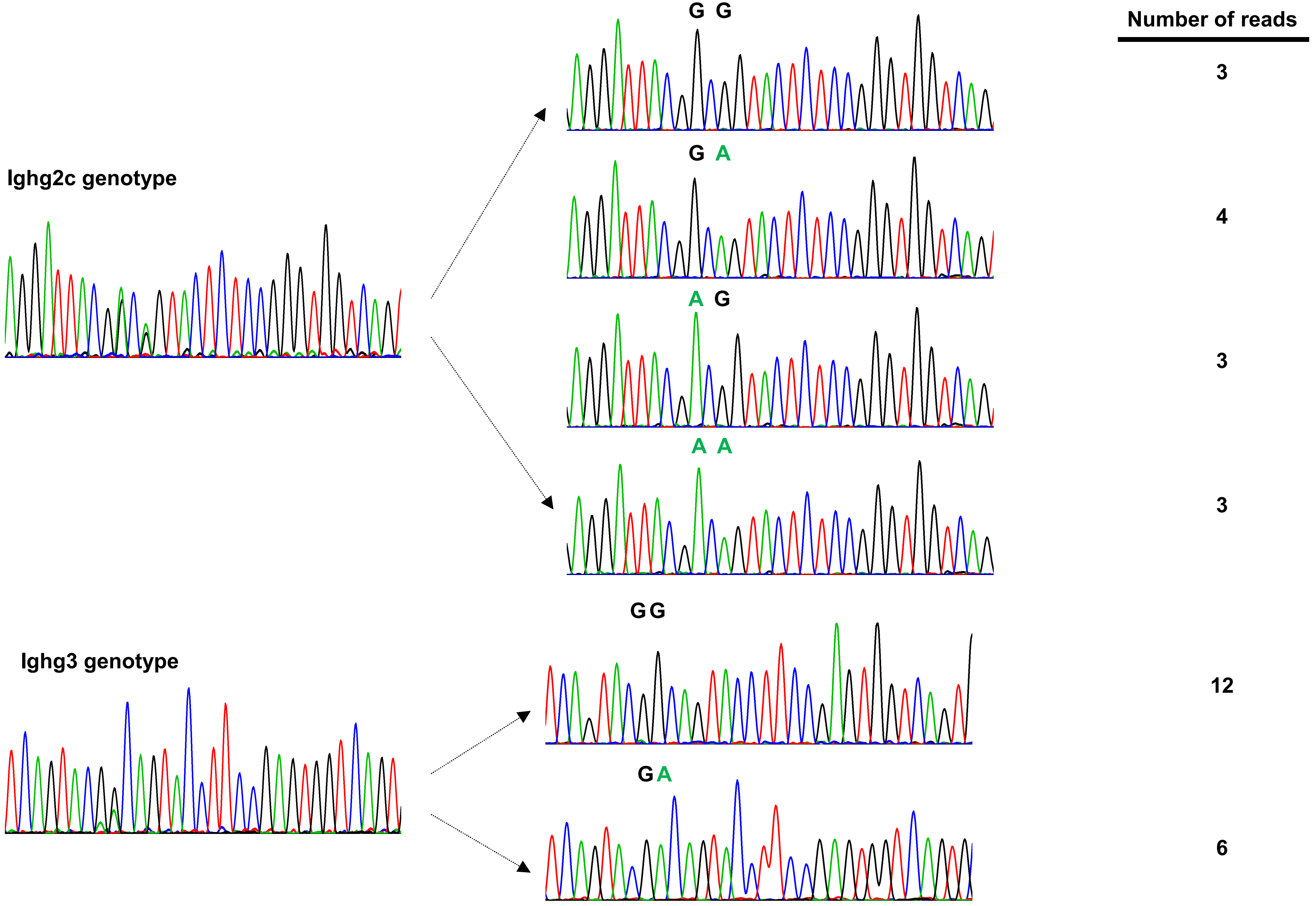


**Supplementary Figure 3. Genotyping of Ighg2c and Ighg3 gene for the pup#11.** This engineered mouse appears to mosaicism for these two *loci*. For Ighg2c, all possible sequences were observed at an almost equal ratio including GG, GA, AG, and AA combinations. For Ighg3, it is estimated that the 'GA' genotype was further converted into 'GG' one at the two or later cell stages.

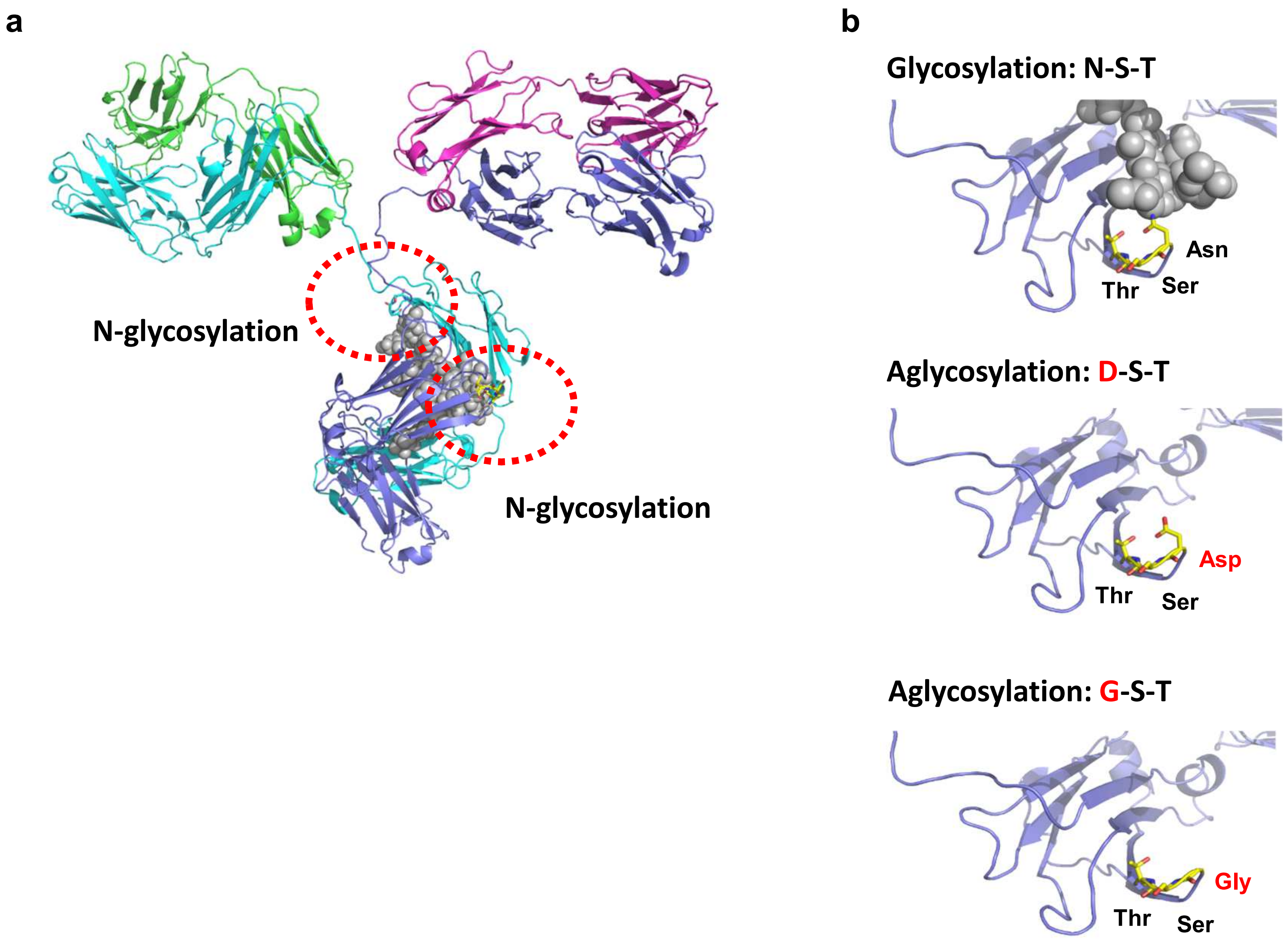


**Supplementary Figure 4. Structural analysis of IgGs to select an intermediate founder.** (a) N-glycosylation site of the asparagine residue (Asn) in the immunoglobulin antibody (IgG2b: PDB 1IGT) was marked with dotted circles. Wild-type IgG2 protein structure shows a conserved motif of N-S-T with the N-glycosylation to the Asn residue. (b) Hypothetical IgG2 structure of the mutant D-S-T and G-S-T antibody without N-glycosylation. The G-S-T motif results in a structural alteration in the β-sandwich fold.

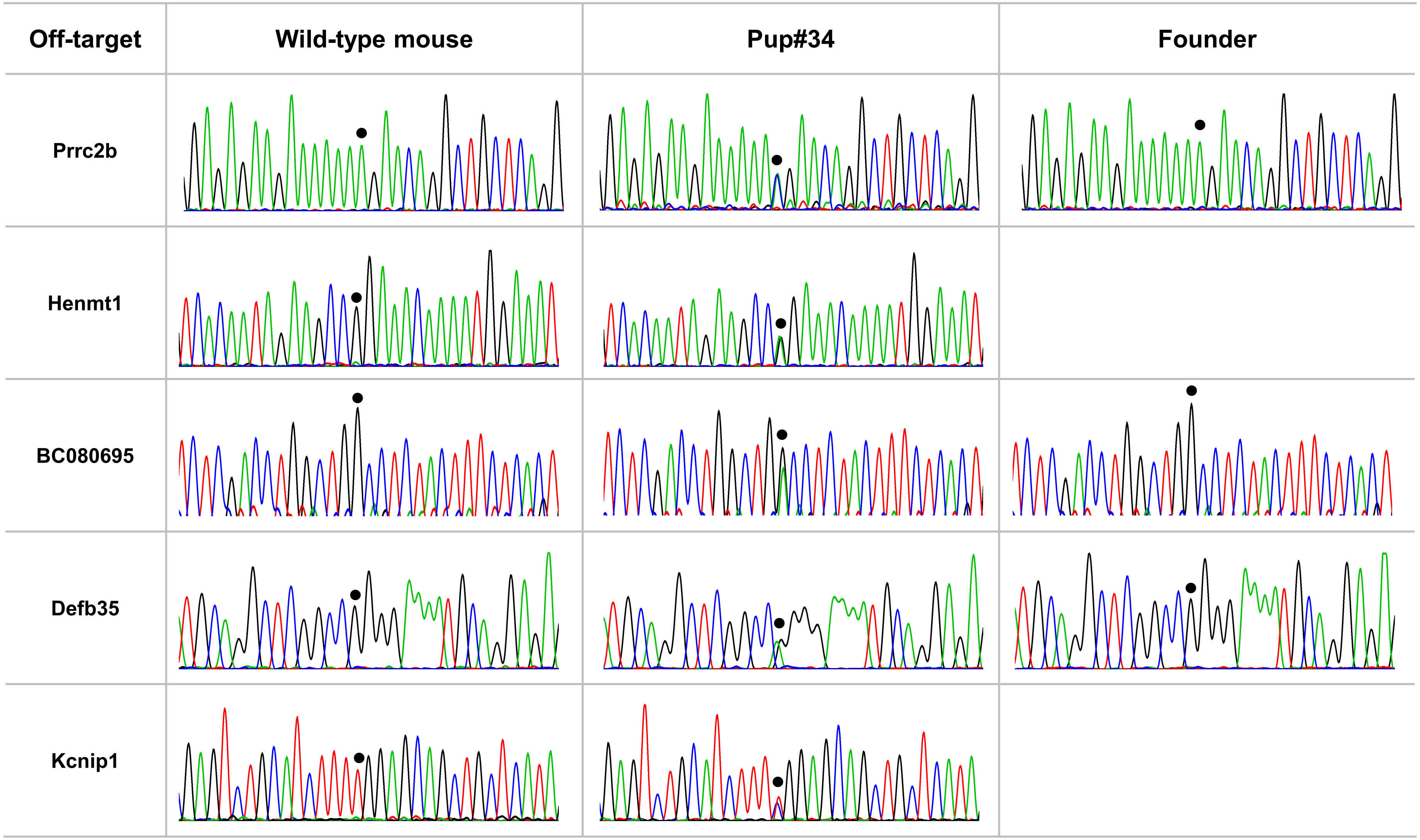


**Supplementary Figure 5. Recovery of off-target mutations in the founder mouse.** The Pub#34 mouse was subjected to several rounds of back-cross with wild-type mice and selection. A founder mice was finally established in which mutations at the five off-target sites were restored into wild-type sequences. The mutation sites were marked with closed circles.

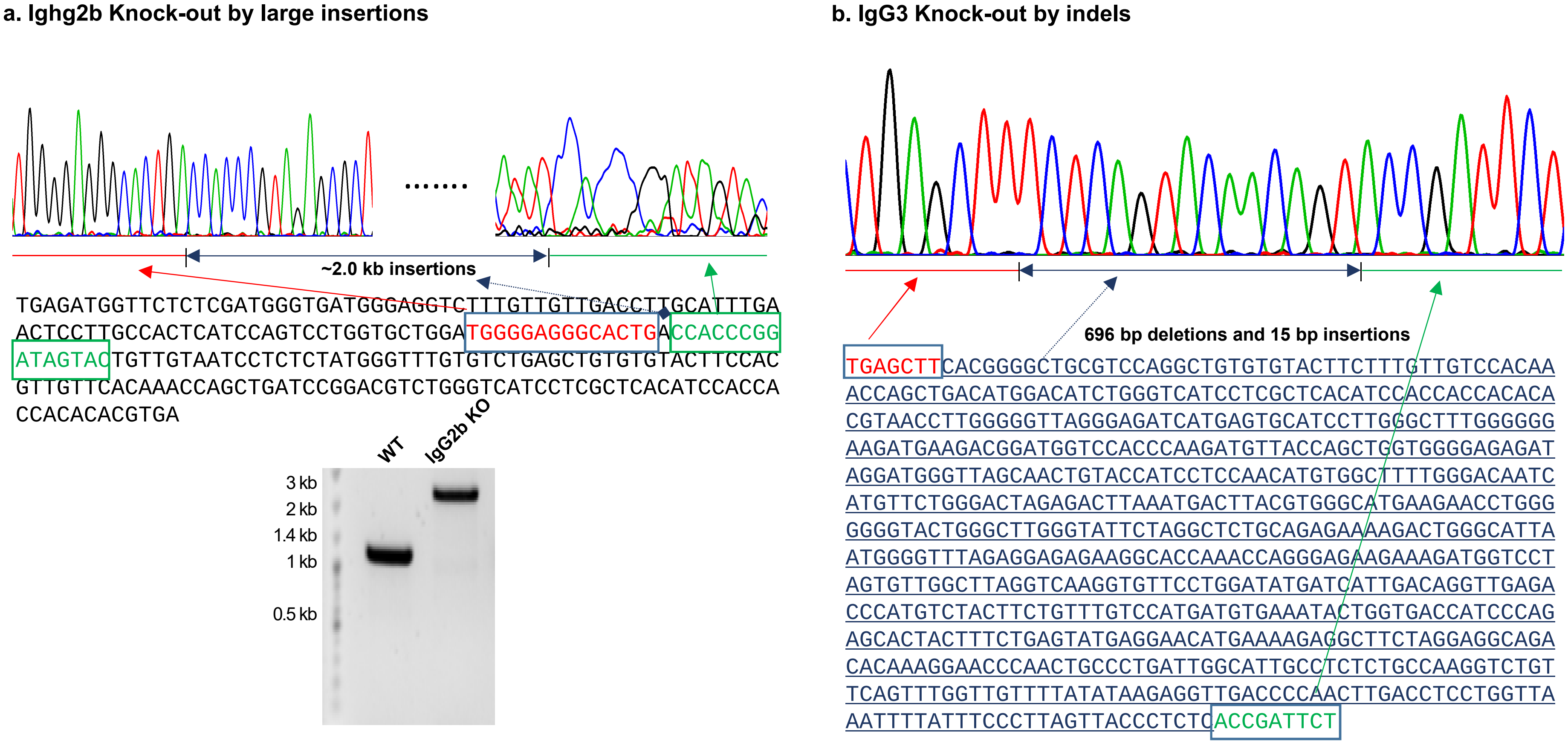


**Supplementary Figure 6. Mutation sequences of a mouse with knock-out at Ighg2b and Ighg3 genes.** (a) A large insertion of *ca.* 2-kb was observed for Ighg2b gene, which was confirmed by Sanger sequencing and PCR analyses. (b) Indel mutations comprising a 696-bp deletion and a 15-bp insertion was identified in the Ighg3 locus. A mouse with a heterozygous insertion at Ighg2b and indels at Ighg3 genes were bred into a homozygous mutant mouse. The homozygous mutant mouse showed a failure in the production of IgG subclass of IgG2b and IgG3 (please see Fig. 3a).

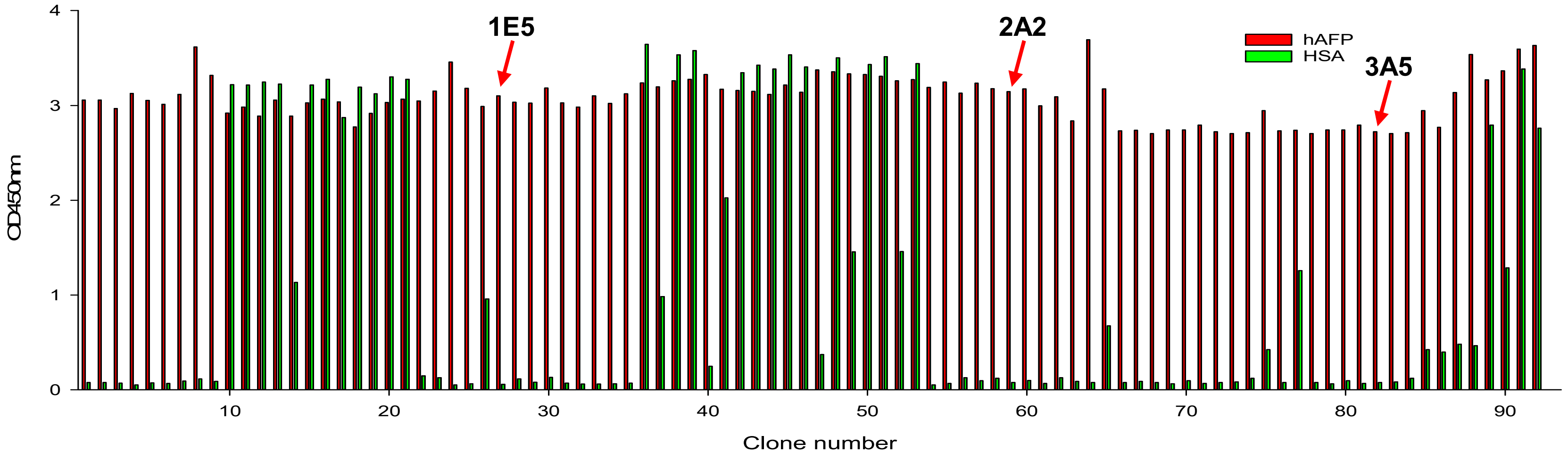


**Supplementary Figure 7. Selection of hybridoma clones that produce highly specific antibodies against hAFP.** Ninety two hybridoma clones that show a high avidity against hAFP was selected among all hybridoma clones. The 92 clones were again investigated in terms of binding affinity against human serum albumin. Three hybridoma clones (1E5, 2A2, and 3A5) were finally selected that show stable expression of IgG and a specific affinity toward hAFP.

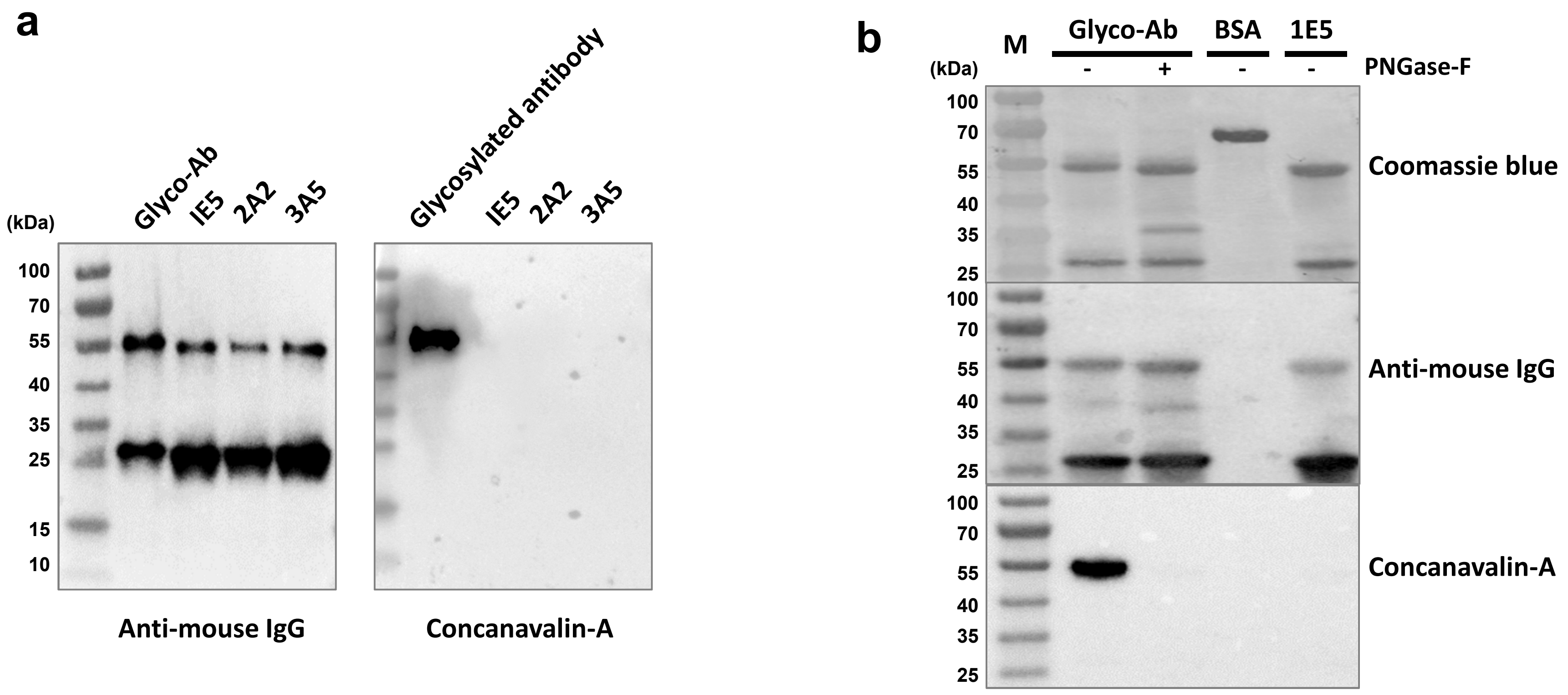


**Supplementary Figure 8. Confirmation of aglycosylation of monoclonal antibodies produced from the three established clones.** (a) In the left gel image, a conventional antibody and the three monoclonal antibodies all show bands of a large subunit and a small subunit, when traced by an anti-mouse IgG antibody. In the right image gel, all the engineered monoclonal antibodies did not bind to Concanavalin-A lectin, whereas the large subunit of a conventional antibody strongly binds to the lectin. (b) 1E5 monoclonal antibody was further confirmed for the absence of N-glycans through lectin blot analysis using Concanavalin-A.

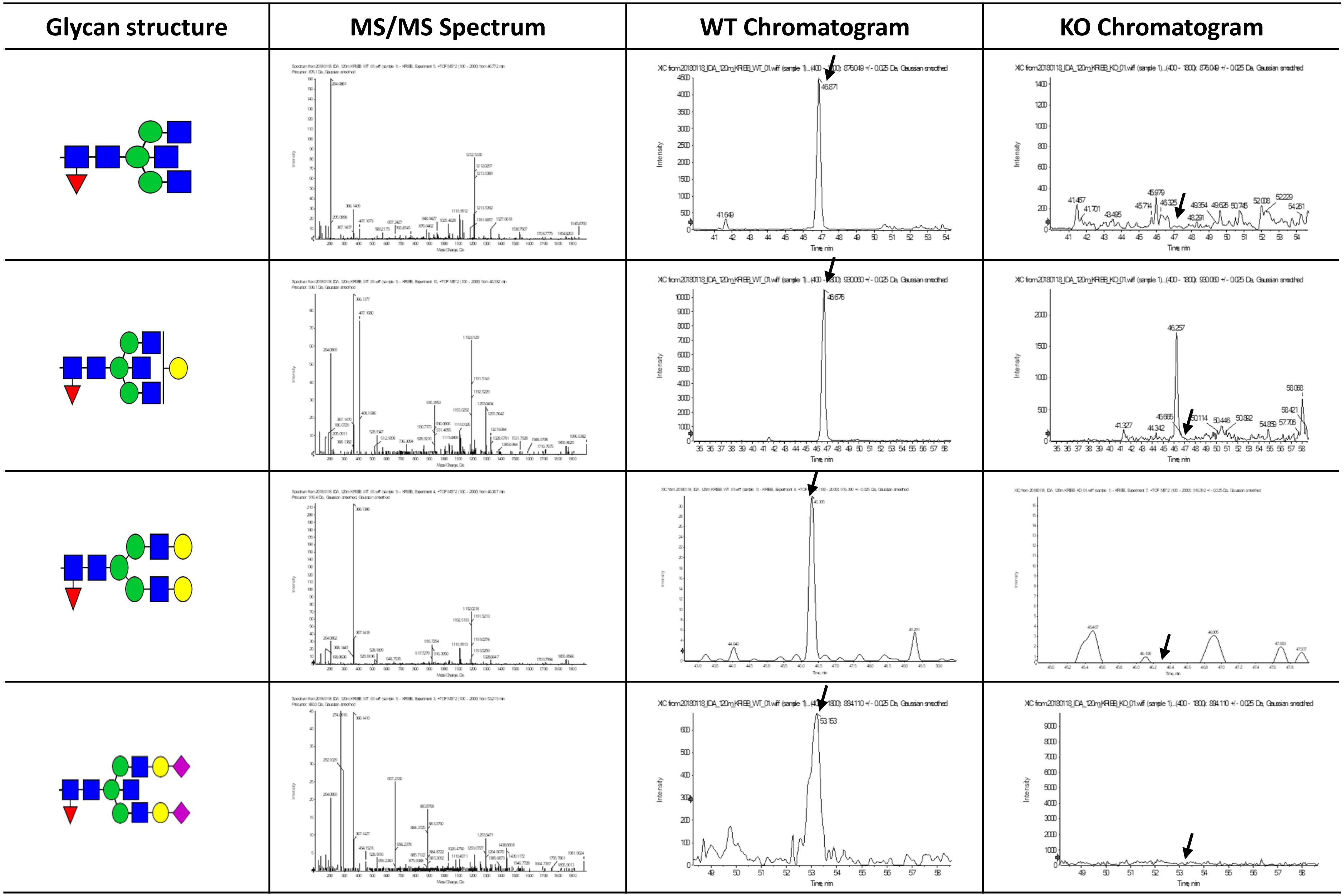


**Supplementary Figure 9. Confirmation of aglycosylation of hAFP by MS/MS analysis.** Representative glycan structures with a peptide sequence of VNFTEIQK analyzed by tandem MS are presented with MS/MS spectra and chromatograms. The absence of fucosylated glycoform in FUT8$^{-/-}$ cells can be confirmed with an absence of peaks found in chromatograms of wild-type samples at the same retention time .

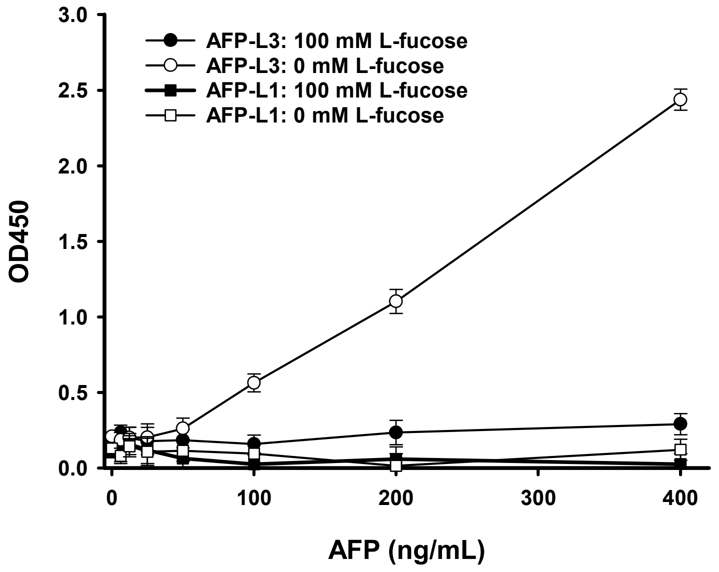


**Supplementary Figure 10. Inhibition of the interaction of AFP-L3 and lectins by L-fucose.** The treatment of L-fucose at 100 mM inhibited the binding of the lectin to core-fucose of AFP-L3, indicating the AFP-L3 specific binding of the lectin.

**Supplementary Table 1**. List of primer pairs for genome engineering

| Target gene | | Analysis primer (5'→3') |
|---|---|---|
| IgG subclass | Ighg1 | F: GCAGCACCAAGGTGGACAAG<br>R: GTGCTGGGTGTGGCAGTGTA |
| | Ighg2b | F: CTCCTAACTCCGAGGGTGGA<br>R: GAGATGGTTCTTCCGATGGG |
| | Ighg2c | F: ACCATCCGTCTTCATCTTCC<br>R: TGTTGTTGACCTTGCATTTG |
| | Ighg3 | F: CTGGTAACATCTTGGGTGGA<br>R: TGAGATGGTTCTCTCGATGG |
| Fut8 (α1,6-fucosyltransferase 8) | Exon 9 | F: ACCAGTGTCAATGCGAGCAT<br>R: TTTCAAGGGCCAGGAAGACT |
| | Exon 11 | F: GTGAAAGGTGGGAGGAGGGT<br>R: TCCAGATGATTCTCATGCATGCT |

**Supplementary Table 2.** Sequence information of primers used for ARMS-PCR

| Primer name | Primer sequence(5'→3') | PCR product size (bp) | | |
|---|---|---|---|---|
| | | Control | A | G |
| Ighg 1_ARMS_outer_F | TCCCAGAAGTATCATCTGTC | 321 | 203 | 147 |
| Ighg 1_ABE+G-ARMS F_14+G | GGAGGAGCAGATCAG | | | |
| Ighg 1_ABE-A-ARMS R_15 | AACGGAAAGTGCTGT | | | |
| Ighg 1_ARMS_outer_R | CTTTGGTTTTGGAGATGGTT | | | |
| Ighg 2b_ARMS_outer F | CTAACCTCGAGGGTGG | 476 | 209 | 296 |
| Ighg 2b_ABE+G-ARMS R_14+G | ATAGAGAGGATTACG | | | |
| Ighg 2b_ABE-A-ARMS R 15 | CCGGATAGTACTGTT | | | |
| Ighg 2b_ARMS_outer_R | GGCGGCAAGATGTATAC | | | |
| Ighg 2c_ARMS_outer F | CATGCGCAGGTAAGTC | 403 | 330 | 102 |
| Ighg 2c_ABE+G-ARMS F _14+G | ATAGAGAGGATTACG | | | |
| Ighg 2c_ABE-A-ARMS R_15 | CCGGAGAGTACTGTT | | | |
| Ighg 2c_ARMS_outer_R | TGTTGTTGACCTTGCATTTG | | | |
| Ighg 3_ARMS_outer_F | CTGGTAACATCTTGGGTGGA | 506 | 212 | 323 |
| Ighg 3_ABE+G-ARMS F_14+G | GTGAAGCTCAGTACG | | | |
| Ighg 3_ABE-A-R_15 | TCGGAAGGTACTGTT | | | |
| Ighg 3_ARMS_outer_R | TTCTTCTTGGACATTTGTT | | | |

**Supplementary Table 3.** List of target sequences for CRISPR targeting

| Purpose of Experiment | Guide RNA ID | Sequence(5'→3') |
|---|---|---|
| FUT8; knock-out | Fut8-e9 | TACTACCTCAGTCAGACAGA |
| | Fut8-e11 | CACCCAGCGAACACTCATCT |
| IgG1; HDR | Ighg1 | TGGGAAGTTCACTGACTGAG |
| IgG1, 2b, 2c, 3; base editing | Ighg2b | TACAACAGTACTATCCGGG TGG |
| | Ighg2c | TACAACAGTACTCTCCGGG TGG |
| | Ighg3 | TACAACAGTACCTTCCGAG TGG |
