# Supplementary material for: Aglycosylated antibody-producing mice for aglycosylated antibody-lectin coupled immunoassay for the quantification of tumor markers (ALIQUAT)
Source: Commun Biol. 2020 Oct 30;3:636. doi: 10.1038/s42003-020-01363-9 (PMC7599229; doi:10.1038/s42003-020-01363-9)
Supplement: Supplementary file 3 — Description of Additional Supplementary Files [file 42003_2020_1363_MOESM3_ESM.pdf]

Description of Additional Supplementary Files

File Name: Supplementary Data 1
Description: Genome-wide off-target analysis for the engineered mouse

File Name: Supplementary Data 2
Description: Source data for the graphs
