# Supplementary material for: Aglycosylated antibody-producing mice for aglycosylated antibody-lectin coupled immunoassay for the quantification of tumor markers (ALIQUAT)
Source: Commun Biol. 2020 Oct 30;3:636. doi: 10.1038/s42003-020-01363-9 (PMC7599229; doi:10.1038/s42003-020-01363-9)
Supplement: Supplementary file 6 — Reporting Summary [file 42003_2020_1363_MOESM6_ESM.pdf]

nature research

Corresponding author(s): Yong-Sam Kim

Last updated by author(s): Aug 26, 2020

Reporting Summary

Nature Research wishes to improve the reproducibility of the work that we publish. This form provides structure for consistency and transparency in reporting. For further information on Nature Research policies, see Authors & Referees and the Editorial Policy Checklist.

Statistics

For all statistical analyses, confirm that the following items are present in the figure legend, table legend, main text, or Methods section.

n/a	Confirmed	
☐	☒	The exact sample size (*n*) for each experimental group/condition, given as a discrete number and unit of measurement
☐	☒	A statement on whether measurements were taken from distinct samples or whether the same sample was measured repeatedly
☐	☒	The statistical test(s) used AND whether they are one- or two-sided<br>*Only common tests should be described solely by name; describe more complex techniques in the Methods section.*
☐	☒	A description of all covariates tested
☐	☒	A description of any assumptions or corrections, such as tests of normality and adjustment for multiple comparisons
☐	☒	A full description of the statistical parameters including central tendency (e.g. means) or other basic estimates (e.g. regression coefficient) AND variation (e.g. standard deviation) or associated estimates of uncertainty (e.g. confidence intervals)
☐	☒	For null hypothesis testing, the test statistic (e.g. *F*, *t*, *r*) with confidence intervals, effect sizes, degrees of freedom and *P* value noted<br>*Give P values as exact values whenever suitable.*
☒	☐	For Bayesian analysis, information on the choice of priors and Markov chain Monte Carlo settings
☒	☐	For hierarchical and complex designs, identification of the appropriate level for tests and full reporting of outcomes
☐	☒	Estimates of effect sizes (e.g. Cohen's *d*, Pearson's *r*), indicating how they were calculated

Our web collection on statistics for biologists contains articles on many of the points above.

Software and code

Policy information about availability of computer code

Data collection	Genome-wide off-target analysis was performed using lofreq, Strelka, and Mutect2 analysis program.
Data analysis	Microsoft Excel, SigmaPlot, MedCalc, SPSS software was used for statistic analysis

For manuscripts utilizing custom algorithms or software that are central to the research but not yet described in published literature, software must be made available to editors/reviewers. We strongly encourage code deposition in a community repository (e.g. GitHub). See the Nature Research guidelines for submitting code & software for further information.

Data

Policy information about availability of data

All manuscripts must include a data availability statement. This statement should provide the following information, where applicable:

- Accession codes, unique identifiers, or web links for publicly available datasets
- A list of figures that have associated raw data
- A description of any restrictions on data availability

The genome sequencing data were deposited at the NCBI Sequence Read Archive (SRA, http://www.ncbi.nlm.nih.gov/sra) under accession number SRP_115978. All other data that support the findings of the present study are available from the corresponding author upon request.

Field-specific reporting

Please select the one below that is the best fit for your research. If you are not sure, read the appropriate sections before making your selection.

☒ Life sciences ☐ Behavioural & social sciences ☐ Ecological, evolutionary & environmental sciences

Life sciences study design

All studies must disclose on these points even when the disclosure is negative.

Sample size	Sample size was estimated based on a previous analysis by Lu et al (Int J Biostat 12, 2016), which resulted in a total of 41 samples with a power of 80%, an alpha level of 0.05, different standardized difference limits of 3.0, and different standardized agreement limits of 0.2.
Data exclusions	No data points were excluded in all experiments conducted in this study.
Replication	All experiments were conducted at least triple times. ICC analysis was conducted to test method reliability.
Randomization	Serum samples used for the ELISA tests were randomly selected from a pool of samples collected from volunteers to validate the designed experiments.
Blinding	Raters were not informed of data obtained from µ-TAS analysis, when undertaking the ALIQUAT analysis.

Reporting for specific materials, systems and methods

We require information from authors about some types of materials, experimental systems and methods used in many studies. Here, indicate whether each material, system or method listed is relevant to your study. If you are not sure if a list item applies to your research, read the appropriate section before selecting a response.

Materials & experimental systems

n/a	Involved in the study	
☐	☒	Antibodies
☐	☒	Eukaryotic cell lines
☒	☐	Palaeontology
☐	☒	Animals and other organisms
☒	☐	Human research participants
☒	☐	Clinical data

Methods

n/a	Involved in the study	
☒	☐	ChIP-seq
☒	☐	Flow cytometry
☒	☐	MRI-based neuroimaging

Antibodies

Antibodies used	All commercial antibodies used in this study were mentioned in Methods section. The engineered antibodies are also described in Methods section.
Validation	Unless specified, antibodies that have been validated by manufacturers or in previous literature for ELISA test, western blot or Immunofluorescense were used.

Eukaryotic cell lines

Policy information about cell lines

Cell line source(s)	Wild-type cell lines were obtained from ATCC. Knock-out cell lines were generated in house from the cell lines obtained from ATCC
Authentication	None of the cell lines used were authenticated.
Mycoplasma contamination	All cell lines are frequently tested for mycoplasma contamination. Cell lines used in this study were verified to be mycoplasma-free before undertaking any experiments with them.
Commonly misidentified lines (See ICLAC register)	No commonly misidentified cells were used.

Animals and other organisms

Policy information about studies involving animals; ARRIVE guidelines recommended for reporting animal research

Laboratory animals	C57BL/6J, ICR and Balb/c (The Jackson laboratory) mice were used in this study. Males at over 8 weeks and females at over 4 weeks of age were used. Zygotes were obtained from C57BL/6J mice and ICR females were used for recipients. Engineered C57BL/6J mice were back-crossed with Balb/c mice.
Wild animals	This study did not involve wild animals.

Field-collected samples	This study did not involve field-collected samples.
Ethics oversight	Procedures for the use and care of animals were reviewed and approved by the Institutional Animal Care and Use Committee (IACUC), KRIBB.

Note that full information on the approval of the study protocol must also be provided in the manuscript.
